# Primary culture and characterization of human upper limb muscle satellite cells: an experimental study

**DOI:** 10.12701/jyms.2026.43.39

**Published:** 2026-06-12

**Authors:** Young-Ju Lim, Min-Jung Ma, Wansuk Son, Seunghyun Kang, Joo-Hee Choi, Bum-Jin Shim, Min-Soo Seo, Wook-Tae Park

**Affiliations:** 1 Department of Orthopedic Surgery, Yeungnam University Medical Center, Yeungnam University College of Medicine, Daegu, Korea; 2 Laboratory of Veterinary Tissue Engineering, College of Veterinary Medicine, Kyungpook National University, Daegu, Korea; 3 Preclinical Research Center, Daegu-Gyeongbuk Medical Innovation Foundation, Daegu, Korea

**Keywords:** Cell differentiation, Muscle, Myogenesis, Satellite cells, Skeletal muscle

## Abstract

**Background:**

Human muscle satellite (stem) cells (MuSCs) are essential for investigating muscle physiology, regeneration, and disease mechanisms. Primary cultures derived directly from human tissues offer a more physiologically relevant model than immortalized cell lines. However, the isolation and characterization of MuSCs from human upper limb tissues are limited. Therefore, this study aimed to establish and characterize a primary culture system for MuSCs obtained from human upper limb muscle tissue.

**Methods:**

Human muscle tissues were obtained from upper limb surgical specimens. Muscle samples were mechanically and enzymatically dissociated to isolate muscle-derived cells, which were cultured under standard growth conditions. Cell morphology and proliferation were monitored during the culture period. Myogenic characteristics were assessed by examining the expression of muscle-specific markers including myogenic regulatory factors and structural proteins. Additionally, myogenic differentiation capacity was evaluated by inducing differentiation and analyzing the formation of multinucleated myotubes.

**Results:**

Primary MuSCs were isolated from human upper limb tissues and expanded in vitro. The cultured cells exhibited a typical spindle-shaped morphology and demonstrated significant proliferative capacity. Characterization confirmed the expression of myogenic markers, indicating the presence of muscle-derived precursor cells. Following induction of differentiation, the cells formed multinucleated myotube-like structures and expressed muscle proteins associated with differentiation, highlighting their potential for myogenic differentiation.

**Conclusion:**

This study established a reliable protocol for isolating and culturing MuSCs from human upper limb tissues. Cultured cells displayed typical myogenic characteristics and differentiation capacity, indicating that this model could be a valuable platform for studying human muscle biology and potential therapeutic applications.

## Introduction

Skeletal muscle is a highly specialized and dynamic tissue that is crucial for voluntary movement, posture maintenance, and whole-body metabolic homeostasis [1-3]. It constitutes approximately 40% of the total body mass and plays vital roles in locomotion, glucose metabolism, energy balance, and systemic physiological regulation [4,5]. The regenerative capacity of skeletal muscle primarily emanates from a population of resident muscle stem cells, known as satellite (stem) cells, located between the basal lamina and sarcolemma of muscle fibers [6,7]. In response to injury or physiological stimuli, satellite (stem) cells are activated, proliferate, and differentiate into myoblasts, which subsequently fuse to form multinucleated myotubes, effectively restoring muscle structure and function [8,9].

Owing to these unique regenerative properties, skeletal muscle has been a focal point in research on tissue regeneration, aging, muscle-wasting diseases, and metabolic disorders [9-11]. In particular, in vitro culture systems using skeletal muscle cells are essential for investigating myogenesis, cellular signaling pathways, and therapeutic strategies [12,13]. Primary cultures of human skeletal muscle cells are especially valuable because they maintain the physiological characteristics, genetic background, and heterogeneity of native human tissues [6,14]. Compared to immortalized or animal-derived cell lines, primary human muscle cells offer a more relevant experimental model for translational research, encompassing drug screening, regenerative medicine, and cell-based therapies [14,15].

Previous studies have developed methods for isolating and culturing human skeletal muscle cells from tissue biopsies and characterizing their proliferative and myogenic differentiation capacities [6,11,14]. However, most studies have focused on skeletal muscles derived from the quadriceps, gastrocnemius, and tibialis anterior muscles [16,17]. This is primarily because of their larger mass and accessibility for biopsies [18-20]. Consequently, much of our understanding of human skeletal muscle biology stems from these lower limb-derived cells [21,22]. In contrast, human muscle satellite cells were isolated from the flexor digitorum superficialis muscle obtained from the forearm during surgical procedures in the present study.

Tissues from upper limb skeletal muscle have been underexplored in primary culture studies. Nonetheless, the upper limb muscles represent a clinically relevant and accessible tissue source, particularly in orthopedic, reconstructive, and trauma-related surgical procedures, where muscle specimens often arise as surgical byproducts [23,24]. Furthermore, upper and lower extremity muscles differ in their physiological, histological, and compositional characteristics. Compared with the lower extremity muscles, the upper extremity muscles have a higher proportion of type II fibers and exhibit differences in metabolic and fat oxidation capacity, rendering them more dependent on anaerobic metabolism [25-27]. Therefore, investigating skeletal muscle cells derived from human upper limbs could yield valuable insights into muscle biology and enhance the applicability of in vitro muscle models [28,29].

Establishing a reliable primary culture system using tissues from human upper limb skeletal muscle is crucial for expanding experimental platforms and improving the relevance of muscle research [30]. Moreover, characterization of these cells, including their morphology, proliferative capacity, myogenic marker expression, and differentiation potential, is vital for validating their utility as in vitro models [30,31]. This is particularly important for future applications in regenerative medicine, disease modeling, and the development of therapeutic strategies for muscle-related disorders [7,32].

In the present study, we isolated skeletal muscle-derived cells from human upper limb tissues and established a primary culture system under standard in vitro conditions [33]. The cultured cells were characterized by analyzing their morphological features, proliferative behavior, and expression of key myogenic markers, as well as their capacity to undergo myogenic differentiation and form multinucleated myotubes. Ultimately, this study provides a foundational framework for utilizing human upper limb skeletal muscle-derived cells as physiologically relevant models for muscle biology and therapeutic research.

## Methods

**Ethics statement:** This study was approved by the Institutional Review Board (IRB) of the Yeungnam University Hospital (IRB No: 2024-03-016-001). Written informed consent was obtained from all tissue donors prior to sample collection.

### 1. Human upper limb muscle satellite (stem) cell isolation and culture

Human skeletal muscle tissues were isolated from upper limb surgical specimens with donor consent following IRB-approved guidelines. Tissues from human upper limb muscles were obtained from the upper limb surgical specimens of donors without known neuromuscular disorders, muscle injuries, or degenerative muscle diseases. Only macroscopically normal muscle tissue was used for satellite cell isolation. The samples were immediately transferred to sterile phosphate-buffered saline (PBS) containing 1% penicillin-streptomycin and processed within 2 hours of excision.

The muscle tissues were washed multiple times with PBS to eliminate blood and debris; subsequently, they were minutely chopped into fragments (approximately 1–2 mm³) using sterile scissors. The fragments were enzymatically dissociated in a digestion buffer containing 0.2% collagenase type II at 37°C for 30 to 60 minutes with gentle agitation. After digestion, the cell suspension was filtered through a 70-μm cell strainer to remove undigested tissue.

The filtrate was centrifuged at 300×*g* for 5 minutes, and the resultant cell pellet was resuspended in Dulbecco’s Modified Eagle’s Medium supplemented with 10% fetal bovine serum, 10% horse serum, 1% penicillin-streptomycin, and 1% GlutaMAX (Thermo Fisher Scientific, Waltham, MA, USA). Thereafter, the cells were plated on culture dishes precoated with collagen type I and incubated at 37°C in a humidified environment with 5% CO_2_.

After 24 hours, nonadherent cells were removed by replacing the medium, and adherent cells were cultured with medium changes every 2 to 3 days. When the cells reached approximately 70% to 80% confluence, they were passaged using trypsin-ethylenediaminetetraacetic acid (EDTA) for further expansion. Cultured cells were used for subsequent experiments between passage two and passage 15 (P15).

### 2. Cumulative population doubling level analysis

To evaluate the proliferative capacity of human upper limb muscle satellite (stem) cells (MuSCs), the cumulative population doubling level (CPDL) was calculated during serial passaging. Cell counts were obtained at each passage using a hemocytometer or an automated cell counter, and population doubling (PD) for each passage was determined using the following formula:


$$PD=\frac{\ln\left(N_{f}/N_{i}\right)}{\ln2}$$

where *N_f_* represents the number of harvested cells and *Ni* denotes the number of initially seeded cells. CPDL was derived by summing the PD values from each passage. Cells were continuously cultured and passaged up to P15, and CPDL values were plotted against passage number to assess long-term proliferative potential.

### 3. Reverse transcription polymerase chain reaction

Total RNA was isolated from cultured human upper limb MuSCs using TRIzol reagent (BioScience Technology Laboratory Inc., Bozeman, MT, USA), according to the manufacturer’s instructions. The concentration and purity of extracted RNA were determined using a microplate reader.

For complementary DNA (cDNA) synthesis, 2 μg of total RNA was reverse transcribed using a reverse transcription kit following the manufacturer’s protocol. The resulting cDNA served as a template for the polymerase chain reaction (PCR) analysis of gene expression. Table 1 lists the specific primers for reverse transcription PCR (RT-PCR). The PCR products were separated on a 1% agarose gel. RNA expression levels were compared after normalization to endogenous glyceraldehyde-3-phosphate dehydrogenase mRNA levels.

### 4. Fluorescence-activated cell sorting analysis

To assess cell surface marker expression, cultured human upper limb MuSCs were analyzed using flow cytometry. Cell staining followed the manufacturer’s protocol for cell surface immunofluorescence (BioLegend, San Diego, CA, USA). Briefly, cells were washed twice with PBS and detached using 0.25% trypsin-EDTA. After collection and washing with PBS, approximately 1×10^5^ cells were resuspended for antibody staining with PBS. Flow cytometry was performed using passage five (P5) MuSCs, and cells not stained with the primary antibody were used as controls. The cells were incubated with antibodies against human cell surface markers, including cluster of differentiation (CD)45, CD34, CD90, and CD105 (BioLegend) for 30 minutes at 4°C in the dark. After incubation, the cells were washed twice with PBS to remove unbound antibodies. Flow cytometric analysis was performed using a NovoCyte Advanteon flow cytometer (Agilent Technologies, Santa Clara, CA, USA), and the data were analyzed using NovoExpress software (Agilent Technologies).

### 5. Senescence-associated β-galactosidase staining

Cellular senescence in human upper limb MuSCs was evaluated using senescence-associated β-galactosidase (SA-β-gal) staining. Cells were analyzed at P15 and P5. Briefly, the cells were seeded in culture plates and allowed to reach approximately 70% to 80% confluence. After two washes with PBS, the cells were fixed for 10 to 15 minutes at room temperature. After fixation with 4% paraformaldehyde, the cells were washed with PBS and incubated with β-galactosidase staining solution per the manufacturer’s instructions at 37°C in a CO_2_-free incubator overnight. Senescent cells were identified by blue staining under a light microscope, and the proportion of SA-β-gal-positive cells was determined by counting stained and unstained cells in randomly selected fields.

### 6. Jenner-Giemsa staining

Jenner-Giemsa staining was performed to assess myotube formation during myogenic differentiation. Briefly, cells were fixed with methanol at room temperature and stained with Jenner staining solution (Sigma-Aldrich, St. Louis, MO, USA), followed by Giemsa staining according to the manufacturer’s instructions. After 10 minutes, the cells were washed twice with distilled water and air dried. Stained cells were observed under a light microscope (Nikon, Tokyo, Japan). Protein-rich myotubes appeared as dark purple structures, and the nuclei were stained pink. For quantitative analysis, random microscopic fields were selected for each experimental condition and all visible myotubes within each field were evaluated. The total number of nuclei and number of nuclei incorporated into the myotubes were counted in each field. The fusion index was determined by calculating the percentage of nuclei incorporated into elongated multinucleated myotubes [34,35]. Morphological analysis was performed using ImageJ software (version 1.54d; National Institutes of Health, Bethesda, MD, USA).

### 7. Creatine kinase activity assay

Creatine kinase (CK) activity was measured to evaluate the myogenic differentiation of human upper limb MuSCs. Briefly, cells were washed with PBS, collected, and centrifuged at 1,000×*g* for 15 minutes. Supernatants were collected and subjected to CK activity analysis using a CK assay kit (BioAssay Systems, Hayward, CA, USA), according to the manufacturer’s instructions. The absorbance (optical density, OD) was measured at 340 nm using a VersaMax microplate reader (Molecular Devices, San Jose, CA, USA). The CK activity was calculated using the following equation:


CK(U/L)=OD40 min−OD20 minODcalibrator−ODH2O×150

### 8. Statistical analysis

All experiments were performed in triplicate, and the data are presented as the mean±standard deviation (SD). Statistical analyses were conducted using GraphPad Prism software version 9.0 (GraphPad Software, San Diego, CA, USA). Comparisons between two groups were made using an unpaired Student *t*-test, whereas multiple group comparisons were analyzed using one-way analysis of variance followed by the Tukey *post hoc* test. Statistical significance was set at *p*≤0.05.

## Results

### 1. Isolation and morphological characterization of human muscle satellite (stem) cells from upper limb

**Fig. 1.**
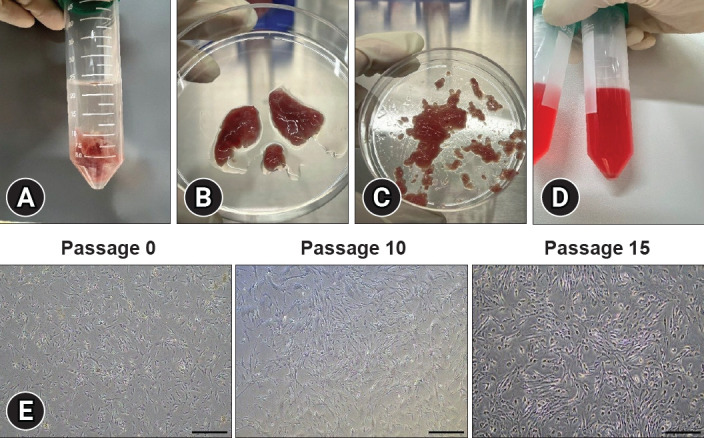
Isolation and morphological characterization of human upper limb muscle satellite (stem) cells (MuSCs). (A) Representative image of freshly obtained human upper limb skeletal muscle tissue. (B) Removal of surrounding connective tissue to enrich muscle components. (C) Mechanical mincing (chopping) of muscle tissue into small fragments. (D) Enzymatic digestion and preparation of a homogeneous cell suspension. (E) Phase-contrast microscopy images of cultured MuSCs at different passages (P0, P10, and P15), exhibiting progressive morphological changes. At P15, cells display increased size and a more flattened morphology, indicating altered cellular conditions during prolonged culture (scale bar=250 μm).

Human upper limb MuSCs were successfully isolated from skeletal muscle tissue obtained from surgical specimens (Fig. 1). Freshly collected muscle tissues were processed (Fig. 1A) and the surrounding connective tissues were carefully removed to enrich the muscle components (Fig. 1B). Purified muscle tissues were then mechanically minced into small fragments (Fig. 1C) and enzymatically digested to generate a homogeneous cell suspension (Fig. 1D). After centrifugation and resuspension, the viable cells were obtained and seeded onto culture dishes. During primary culture (passage zero [P0]), adherent cells were visible within 24 hours of seeding. The cells proliferated gradually and displayed a spindle-shaped morphology. Morphological changes were observed at passage 10 (P10) and P15. Phase-contrast microscopy revealed that at P0, the cells appeared small and scattered but gradually attached to the culture surface (Fig. 1E). By P10, the cells were more elongated and aligned, forming dense layers typical of a spindle-shaped morphology. At P15, although the cells retained their elongated shape, they exhibited an increased size, flattened appearance, and reduced density, suggesting a decline in cellular conditions during prolonged in vitro culture. Overall, human upper limb MuSCs were successfully isolated and expanded from P0 to P15, maintaining a characteristic morphology while exhibiting signs of an altered cellular status at P15.

### 2. Immunophenotypic characterization of human muscle satellite (stem) cells from upper limb

**Fig. 2.**
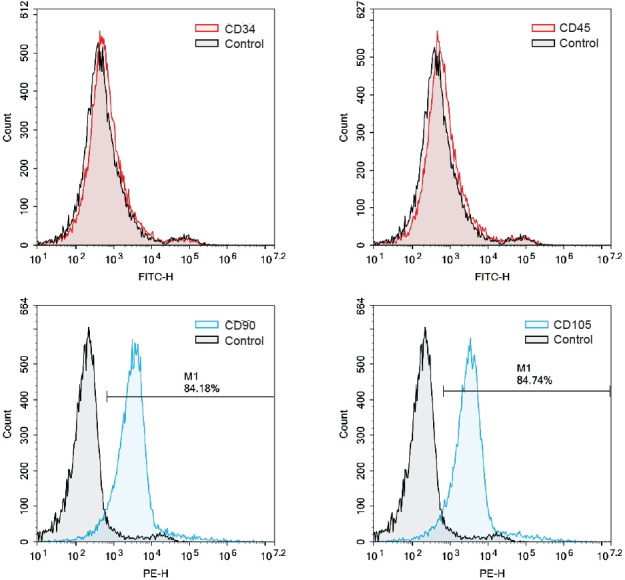
Immunophenotypic analysis of human upper limb muscle satellite (stem) cells via flow cytometry. Flow cytometric analysis reveals the expression of cell surface markers in cultured cells. The cells exhibit low expression of hematopoietic markers (CD34 and CD45) compared with the control, indicating minimal contamination by hematopoietic cells. In contrast, high expression levels of mesenchymal-associated markers (CD90 and CD105) are observed (n=3). These results indicate that the cultured cells display a mesenchymal immunophenotype. CD, cluster of differentiation; FITC, fluorescein isothiocyanate; PE, phycoerythrin; M1, marker 1.

To characterize the immunophenotypic properties of cultured human upper limb MuSCs, we conducted flow cytometry using specific cell surface markers (Fig. 2). The cells showed low expression of hematopoietic markers, including CD34 and CD45, with fluorescence intensity profiles comparable to those of the control group. CD34 is commonly associated with hematopoietic stem cells, while CD45 is a leukocyte marker. The absence of these markers indicates that the cultured cells were generally free from hematopoietic contamination. In contrast, the cells exhibited elevated expression levels of the mesenchymal markers CD90 and CD10^5^, with 84.18%±2.13% and 84.74%±1.87% (mean±SD, n=3) positive cells, respectively. Collectively, these results indicate that the cultured human muscle-derived cells possess a nonhematopoietic, mesenchymal-like immunophenotype, consistent with previously documented characteristics of MuSCs or muscle-derived precursor cell populations.

### 3. Proliferation capacity and cellular senescence of human muscle satellite (stem) cells from upper limb

**Fig. 3.**
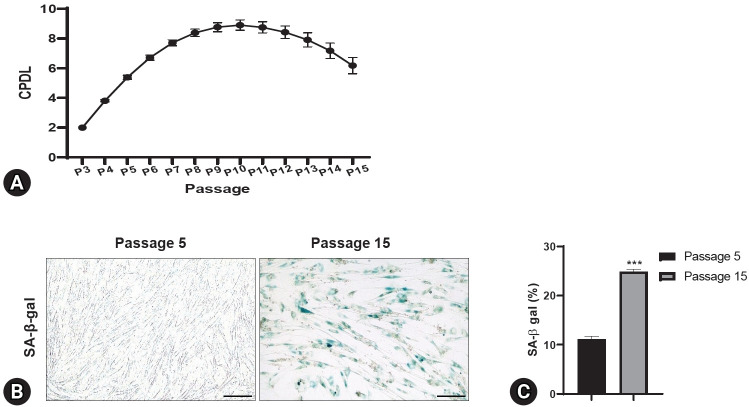
Proliferation and senescence of human upper limb muscle satellite (stem) cells (MuSCs). (A) Cumulative population doubling level (CPDL) of cultured MuSCs across passages (P3–P15). CPDL values increase steadily, peaking around P10, followed by a gradual decline by P15, indicating diminished proliferative capacity. (B) Senescence-associated β-galactosidase (SA-β-gal) staining of MuSCs at P5 and P15. Representative images reveal a marked increase in SA-β-gal-positive (blue-stained) cells at P15 compared with those at P5, suggesting increased cellular senescence with prolonged culture (scale bar=250 μm). Data are presented as the mean±standard deviation. Statistical significance is analyzed using an unpaired Student *t*-test. ***p<0.001.

To evaluate the proliferative capacity of human upper limb MuSCs during long-term in vitro culture, we analyzed the CPDLs across serial passages (Fig. 3A). The CPDL values progressively increased from P3 to P10, indicating active and sustained cell proliferation during the initial expansion phase. The cells exhibited consistent growth patterns, reflecting their in vitro expansion capacity. Nevertheless, after reaching a peak around P10, the CPDL values gradually declined from P11 to P15, suggesting a diminished proliferative capacity with extended culture. This indicates that prolonged passaging may adversely affect the growth potential of MuSCs. To further assess cellular senescence associated with long-term culture, we performed SA-β-gal staining at P5 and P15 (Fig. 3B, 3C). P5 cells exhibited minimal SA-β-gal-positive staining, indicating low levels of cellular senescence and a relatively healthy proliferative state. Conversely, P15 cells showed a marked increase in the number of blue-stained cells, indicating enhanced senescence. Additionally, P15 cells displayed morphological features typical of senescent cells, including an increased size and flattened appearance. These findings suggest that human upper limb MuSCs have a robust proliferative capacity at P5 but gradually lose their proliferative potential and undergo replicative senescence during prolonged in vitro culture. Combined analysis involving CPDL and SA-β-gal staining provides consistent evidence of aging-related modifications in MuSCs during serial passaging.

### 4. Myogenic differentiation of human muscle satellite (stem) cells from upper limb

**Fig. 4.**
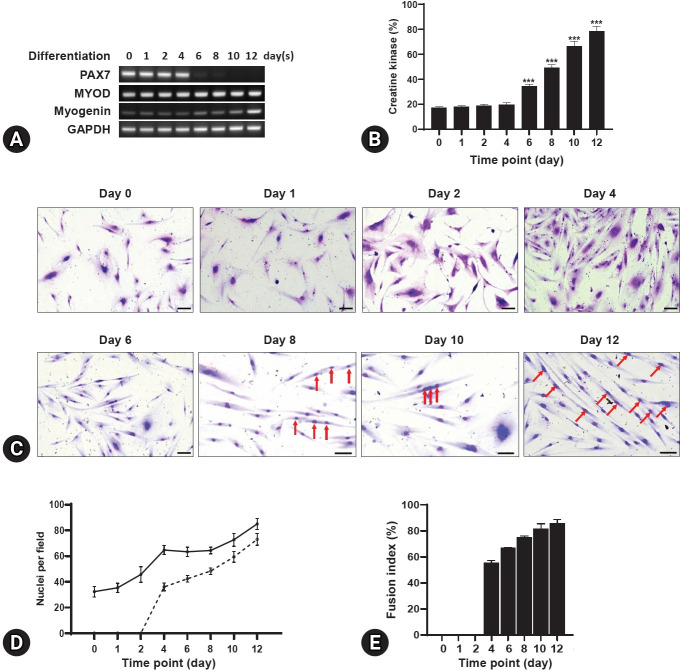
Myogenic differentiation of human upper limb muscle satellite (stem) cell (MuSCs). (A) Reverse transcription polymerase chain reaction analysis of myogenic marker expression during differentiation (0–12 days) demonstrates a time-dependent upregulation of myogenic regulatory factors. (B) Creatine kinase activity measured at various time points demonstrates a progressive increase during differentiation. (C) Jenner–Giemsa staining of MuSCs at specified time points (0–12 days) illustrates morphological alterations from mononuclear cells to elongated, multinucleated myotube-like structures. (D) Quantitative analysis of nuclear distribution during myogenic differentiation. The solid line represents the total number of nuclei per field, whereas the dashed line indicates the number of fused nuclei incorporated into multinucleated myotubes. Red arrows indicate representative myotube-like structures. (E) Fusion index analysis during myogenic differentiation. Fusion index is calculated as the percentage of nuclei incorporated into multinucleated myotubes relative to the total number of nuclei (scale bar=250 μm). Data are presented as the mean±standard deviation. Statistical significance was analyzed using one-way analysis of variance followed by Tukey *post hoc* test. ***p<0.001. PAX7, paired box 7; MYOD, myogenic differentiation 1; GAPDH, glyceraldehyde-3-phosphate dehydrogenase.

To evaluate the myogenic differentiation potential of human upper limb MuSCs, we induced differentiation and analyzed it over time (0, 1, 2, 4, 6, 8, 10, and 12 days; Fig. 4). RT-PCR gene expression analysis revealed progressive upregulation of myogenic markers throughout the differentiation period (Fig. 4A). The expression levels of myogenic regulatory factors increased in a time-dependent manner, indicating activation of the myogenic differentiation program. Notably, early myogenic markers appeared at the initial time points, followed by sustained expression at later stages, suggesting a coordinated transition from myogenic commitment to terminal differentiation. Correspondingly, the activity of CK, a recognized marker of skeletal muscle differentiation, increased significantly over time (Fig. 4B). CK activity began to increase during the early differentiation stages and continued to increase throughout the culture period, peaking at later time points. This increase reflects enhanced metabolic activity and functional maturation of differentiated muscle cells. Jenner-Giemsa staining confirmed these morphological alterations (Fig. 4C). At early time points (0–4 days), the cells exhibited a mononuclear, spindle-shaped morphology typical of undifferentiated MuSCs. As differentiation progressed (6–8 days), the cells elongated and aligned in parallel, indicating the initiation of myogenic fusion. In the later stages (10–12 days), multinucleated myotube-like structures were prominent, indicating successful cell fusion and terminal differentiation. The formation of elongated, protein-rich myotubes is a hallmark feature of skeletal muscle maturation in vitro. Quantitative analysis of nuclear distribution further supported these observations (Fig. 4D). The total number of nuclei per field gradually increased throughout the differentiation period, reflecting continued cell proliferation and survival during early differentiation, followed by sustained cellular accumulation in later stages. In parallel, the number of fused nuclei was minimal at early time points (0–4 days), but markedly increased thereafter, consistent with the onset of myoblast fusion and myotube formation. By 10 to 12 days, the number of fused nuclei prominently increased, indicating the progressive maturation of multinucleated myotubes. Fusion index analysis provided additional quantitative evidence of myogenic differentiation (Fig. 4E). The proportion of nuclei incorporated into myotubes significantly increased in a time-dependent manner, with low values in the early stages and substantial increases in the later stages. This progressive increase demonstrates efficient cell fusion and confirms the transition of MuSCs from proliferative mononuclear cells to mature multinucleated myotubes.

## Discussion

In this study, we established a primary culture system of human upper limb MuSCs derived from surgical skeletal muscle specimens and systematically characterized their biological properties, including proliferative capacity, immunophenotypic profile, cellular senescence, and myogenic differentiation potential. These findings demonstrate that human upper limb muscle tissue can serve as a reliable and practical source of MuSCs for in vitro studies [7,10,36].

Human skeletal muscle-derived cells have been extensively used to investigate muscle regeneration, metabolism, and disease mechanisms [6,10,37,38]. However, previous studies have predominantly relied on biopsies from lower limb muscles, particularly the quadriceps, gastrocnemius, and tibialis anterior muscles, owing to their accessibility and relatively large tissue volumes [18-20]. In contrast, upper limb muscle tissues have been less frequently explored, despite their availability during orthopedic and reconstructive surgeries [39]. This study demonstrated that upper limb muscle tissue can be efficiently processed to yield viable, adherent, and expandable MuSC populations, highlighting its potential as an underutilized yet valuable source of MuSCs for human muscle research.

The isolated MuSCs exhibited a typical spindle-shaped morphology and adhered to the culture surfaces within 24 hours, aligning with established characteristics of activated satellite (stem) cells in vitro. The cells displayed a progressive increase in CPDL, indicating a strong proliferative capacity. However, this proliferative potential declined by P15, accompanied by a notable rise in SA-β-gal-positive cells and morphological changes, such as a larger cell size and flattened appearance. SA-β-gal staining was predominantly observed at P15, indicating that replicative senescence became apparent at this stage during prolonged in vitro expansion. The preservation of these senescence-associated characteristics indicates that the cells maintain intrinsic biological properties reflective of their in vivo origin, thereby supporting their physiological relevance.

Flow cytometric analysis further revealed that the cultured MuSCs were negative for hematopoietic markers (CD34, CD45), suggesting minimal contamination by blood-derived cells. Conversely, the cultured cells exhibited high expression of mesenchymal-associated markers, including CD90 and CD105. This immunophenotypic profile aligns with that of previous reports, indicating that MuSCs and muscle-derived progenitor cells can partially overlap with mesenchymal stem cell-associated markers under in vitro culture conditions. Therefore, the observed expression of mesenchymal markers likely reflects the inherent plasticity and heterogeneity of MuSC populations rather than a distinct mesenchymal lineage [40-42].

Importantly, the myogenic potential of the cultured MuSCs was validated using various complementary approaches. RT-PCR analysis revealed time-dependent upregulation of myogenic regulatory factors during differentiation, indicating activation of the myogenic transcriptional program. These results align with the well-established hierarchical regulation of myogenesis, in which early and late myogenic factors coordinate the transition from proliferation to terminal differentiation. Concurrently, functional maturation of the differentiating cells was evidenced by a significant increase in CK activity over time. As a key enzyme involved in muscle energy metabolism, CK is widely accepted as an indicator of myogenic differentiation. The progressive increase in CK activity suggests that the cells not only initiate myogenic gene expression but also acquire functional properties characteristic of mature muscle cells [43,44].

Morphological analyses further reinforced these findings. Based on CK activity, sequential morphological alterations observed during differentiation, and expression patterns of myogenic markers analyzed by RT-PCR, the differentiation process could be broadly categorized into early (0–4 days), differentiation (6–8 days), and late (10–12 days) stages. Jenner-Giemsa staining showed mononuclear, spindle-shaped cells in the early stage, elongated and aligned cells in the differentiation stage, and multinucleated myotube-like structures in the late stage. These morphological changes are well-known characteristics of skeletal muscle differentiation and reflect the progressive fusion of myoblasts to form mature myotubes. Consistent with these observations, quantitative analysis showed that the total number of nuclei per field gradually increased throughout cell proliferation, suggesting continued cell growth and maintenance during the early phases of culture. In parallel, the number of fused nuclei remained minimal at the initial time points, but increased markedly from the differentiation stage onward, coinciding with the onset of myoblast fusion and myotube formation. Furthermore, the fusion index increased progressively over time, confirming the efficient incorporation of nuclei into multinucleated myotubes during terminal differentiation. The close agreement among the molecular, functional, and morphological findings strongly corroborated the successful myogenic differentiation potential of the isolated MuSCs. Collectively, these findings provide strong evidence that human upper limb MuSCs possess robust proliferative and myogenic differentiation capacities. The use of multiple independent and complementary assays enhances the reliability of these observations and validates this in vitro culture system as a model of human skeletal muscle biology.

Nevertheless, this study has several limitations that must be acknowledged. First, while immunophenotypic analysis was conducted, further validation of satellite stem cell-specific markers such as paired box 7 (PAX7) and myogenic differentiation 1 (MYOD) at the protein level would enhance the identification of MuSC populations. Second, the potential heterogeneity of the isolated cell populations cannot be entirely ruled out, as primary cultures may contain mixed progenitor cell populations. Third, donor-related factors, including age, health status, and anatomical location, potentially influence cellular characteristics and should be considered in future studies.

Future research should focus on assessing the regenerative capacity of these MuSCs in vivo and examining their interactions with extracellular vesicles, growth factors, and biomaterials. Additionally, comparative analyses of MuSCs derived from the upper and lower limbs may yield valuable insights into region-specific differences in skeletal muscle biology and regenerative potential. In conclusion, this study demonstrated that human upper limb skeletal muscle tissue represents a viable and accessible source of MuSCs with considerable proliferative capacity and robust myogenic differentiation potential. This established culture system offers a valuable platform for studying human muscle biology and potentially augments the development of novel therapeutic strategies for muscle-related disorders.

## Figures and Tables

**Table 1.** Specific primers for reverse transcription polymerase chain reaction sequences

Primer	Sequences (5′→3′)
*PAX7*	
Forward	GTGCCCTCAGTGAGTTCGAT
Reverse	GTTTGGCCTTCTTTTCGCCG
*MYOD*	
Forward	TCTCTGCTCCTTTGCCACAA
Reverse	CGAGTGCTCTTCGGGTTTCA
*Myogenin*	
Forward	CGCAGGCTCAAGAAGGTGAA
Reverse	CGCTCGATGTACTGGATGGC
*GAPDH*	
Forward	CGCTGAGTACGTCGTGGAGT
Reverse	GGAGGCATTGCTGATGATCT

*PAX7*, paired box 7; *MYOD*, myogenic differentiation 1; GAPDH, glyceraldehyde-3-phosphate dehydrogenase.
